# Integrative Analysis of Biochemical, Hormonal, and Histopathological Profiles in Thyroid Nodules: A Seven-Year Retrospective Study

**DOI:** 10.3390/biomedicines14010010

**Published:** 2025-12-20

**Authors:** Sergiu-Ciprian Matei, Mervat Matei, Sorin Ursoniu, Anna Laura Maiozzi, Ana Silvia Corlan, Bianca Roxana Natarâş, Flavia Medana Petrașcu, Mihaela Maria Vlad, Diana Szekely, Flavia Zara, Cristina Stefania Dumitru

**Affiliations:** 1 Department of Surgery, “Victor Babeș” University of Medicine and Pharmacy Timisoara, 300041 Timisoara, Romania; matei.sergiu@umft.ro; 2 Doctoral School, “Victor Babeș” University of Medicine and Pharmacy Timisoara, E. Murgu Square, No. 2, 300041 Timisoara, Romania; 3 Department of Endocrinology, Emergency County Hospital, 300723 Timisoara, Romaniavlad.mihaela@umft.ro (M.M.V.); 4 Department of Functional Sciences, Discipline of Public Health, Center for Translational Research and Systems Medicine, 300736 Timișoara, Romania; 5 Faculty of Medicine, “Victor Babeș” University of Medicine and Pharmacy Timișoara, EftimieMurgu Sq. no. 2, 300041 Timisoara, Romania; 6 Department of Internal Medicine II, Discipline of Endocrinology, “Victor Babeș” University of Medicine and Pharmacy, 300041 Timisoara, Romania; 7 Anapatmol Research Center, “Victor Babeș” University of Medicine and Pharmacy Timișoara, 300041 Timisoara, Romania; 8 Department of Biochemistry, “Victor Babeș” University of Medicine and Pharmacy Timișoara, 300041 Timisoara, Romania; 9 Department II of Microscopic Morphology, Discipline of Histology, “Victor Babeș” University of Medicine and Pharmacy Timișoara, E. Murgu Square, No. 2, 300041 Timisoara, Romaniacristina-stefania.dumitru@umft.ro (C.S.D.)

**Keywords:** thyroid disease, biochemical markers, hormonal profile, papillary thyroid carcinoma, retrospective study, risk stratification, thyroid malignancy, thyroid nodules

## Abstract

**Background/Objectives**: Thyroid nodules exhibit substantial histopathological variability, and systemic markers that differentiate benign from malignant patterns remain poorly defined. This study evaluated clinical, biochemical, hormonal, and histopathological characteristics in patients undergoing total thyroidectomy for nodular thyroid disease. **Methods**: A retrospective cohort of 926 patients operated between 2017 and 2024 was analyzed. Patients were classified as: Group 1—benign lesions; Group 2—benign–malignant associations; Group 3—multiple malignant lesions. Demographic, biochemical, hormonal, and histopathological data were assessed using the Kruskal–Wallis and Mantel–Haenszel chi-square tests. Thyroid-specific tumor and autoimmunity markers (calcitonin, thyroglobulin, anti-thyroglobulin antibodies, and thyroid peroxidase antibodies) were not included in the comparative analyses due to their non-uniform availability across the retrospective cohort. **Results**: Most clinical and biochemical parameters showed no significant differences among the three groups, including TSH (*p* = 0.122), FT3 (*p* = 0.560), glycemia (*p* = 0.829), creatinine (*p* = 0.193), fibrinogen (*p* = 0.535), and thyroid dimensions (length *p* = 0.401, width *p* = 0.183, thickness *p* = 0.667, and total thyroid mass *p* = 0.109). Neutrophil count differed in the overall comparison (*p* = 0.021), although absolute differences were small, and lymphocyte counts were modestly lower in patients with multiple malignant lesions compared with benign disease (*p* = 0.009). Comorbidities and BMI were similarly distributed across groups (all *p* > 0.05). Overall, routinely available clinical, biochemical, and hormonal parameters demonstrated limited discriminatory value between patients with different histopathological patterns. **Conclusions**: Standard clinical, biochemical, and hormonal markers showed minimal ability to reflect underlying histopathological patterns in patients with thyroid nodules, underscoring their limited utility for preoperative risk stratification.

## 1. Introduction

Thyroid nodules are frequently encountered in clinical practice, particularly among women and older individuals, and are often asymptomatic with a generally low risk of malignancy [1,2]. Nevertheless, approximately 10–15% of nodules are malignant, underscoring the need for accurate diagnostic tools and proper risk stratification to guide therapeutic decisions [3]. Clinicians play a crucial role in distinguishing benign from malignant nodules using approaches that are both minimally invasive and cost-effective.

The higher prevalence of nodules in women has been linked to hormonal influences, especially estrogens, which may promote thyroid cell proliferation [4]. Additional risk factors include iodine deficiency, prior radiation exposure, family history of thyroid disease, and several environmental or dietary contributors [5,6]. Childhood radiation exposure is a well-established determinant of thyroid nodule development, and individuals treated with head, neck, or chest radiation require long-term surveillance due to their increased risk of thyroid malignancy [7,8]. Genetic predispositions—such as *RET*, *BRAF*, or *RAS* mutations—further contribute to the likelihood of malignant transformation [9].

Most thyroid nodules are benign and include lesions such as multinodular goiter, thyroid adenoma, or simple cysts [10]. These nodules typically require only clinical and ultrasound monitoring rather than invasive treatment [11]. In contrast, malignant nodules constitute a smaller proportion of cases and comprise papillary thyroid carcinoma (PTC), follicular thyroid carcinoma (FTC), medullary thyroid carcinoma (MTC), and, more rarely, anaplastic thyroid carcinoma (ATC) [12]. PTC is the predominant form and generally presents with an excellent prognosis when identified early [13]. FTC also carries a favorable outlook with adequate treatment, whereas MTC is frequently associated with syndromic conditions such as Multiple Endocrine Neoplasia. ATC remains the most aggressive subtype, with rapid progression and poor therapeutic response [14,15].

Clinical evaluation of thyroid nodules begins with a thorough medical history and physical examination, focusing on symptom duration, family history, radiation exposure, and compressive symptoms such as dysphagia or hoarseness [16,17,18]. Ultrasound (US) constitutes the cornerstone of diagnostic assessment, enabling detailed characterization of size, echogenicity, margins, calcifications, and other suspicious features associated with malignancy [19,20,21]. These findings form the basis of the American Thyroid Association (ATA) risk stratification systems, which help determine the need for additional diagnostic procedures [22].

Fine-needle aspiration (FNA) biopsy is the primary method for cytological evaluation of nodules that meet specific sonographic criteria [23,24]. Cytology is classified using the Bethesda System, which standardizes reporting into six diagnostic categories, including indeterminate groups that may require further molecular analysis [19,25]. Molecular testing has emerged as a valuable adjunct in assessing the risk of malignancy in indeterminate nodules by evaluating gene mutations and other oncogenic markers [26,27].

Beyond imaging and cytology, biochemical and hormonal parameters remain integral to the overall assessment of thyroid nodules. Thyroid-stimulating hormone (TSH), free thyroxine (T4), and free triiodothyronine (T3) help evaluate thyroid function and may correlate with malignancy risk [10,15]. Routine laboratory tests further contribute to the understanding of systemic or metabolic alterations that may accompany thyroid nodular disease, emphasizing the importance of a comprehensive, multimodal evaluation [28].

Management strategies vary according to nodule size, sonographic risk, and cytological or molecular findings [17]. While benign, asymptomatic nodules typically require periodic monitoring, nodules with suspicious or malignant features often necessitate surgical treatment, including lobectomy or total thyroidectomy, followed by radioactive iodine therapy in selected cases [14,19,29,30].

Given the high prevalence of thyroid nodules and the multifactorial nature of their evaluation, an integrated approach combining clinical, biochemical, hormonal, and histopathological data is essential. The present study provides a comprehensive seven-year retrospective assessment of patients with thyroid nodules, focusing on the prevalence of different histopathological types, their overlap within the same gland, and the associated biochemical and hormonal variations. Our objective was to investigate how these parameters differ across benign–benign, benign–malignant, and malignant–malignant patterns, and to identify potential diagnostic indicators that may support personalized therapeutic decision-making.

## 2. Materials and Methods

### 2.1. Study Design, Setting, and Population

This study is an observational, retrospective cohort analysis. We reviewed the medical records of 1097 patients followed in the Endocrinology Department of the “Pius Brînzeu” Emergency County Hospital, Timișoara, Romania, who were diagnosed with thyroid nodules and subsequently admitted and surgically treated in the 1st Surgical Department of the same hospital between January 2017 and January 2024. All patients diagnosed with thyroid nodules during the defined period were initially considered for eligibility. Clinical charts, paraclinical investigations, surgical reports, and pathology records were examined in detail. The study protocol was approved by the institutional ethics committee (REC no. 481/12.08.2024), and all procedures were conducted in accordance with the Declaration of Helsinki.

### 2.2. Data Collection and Variables

The following variables were collected for statistical analysis:

- Demographic data: age, sex, and area of residence (urban/rural);
- Clinical data: body mass index (BMI), presence of chronic comorbidities (diabetes mellitus, arterial hypertension), and association of other benign or malignant tumors;
- Laboratory parameters: complete blood count (CBC) including red blood cell (RBC) count, white blood cell (WBC) count with differential (neutrophils, lymphocytes, eosinophils, basophils, monocytes), platelet count (PLT), erythrocyte sedimentation rate (ESR), and fibrinogen;
- Hormonal and biochemical profile: free triiodothyronine (FT3), free thyroxine (FT4), thyroid-stimulating hormone (TSH), total protein (TP), blood glucose (glycemia), and creatinine;
- Surgical data: type of procedure (thyroid lobectomy, subtotal thyroidectomy, or total thyroidectomy);
- Pathological parameters: overall specimen dimensions (length, height, width, in centimeters), specimen weight (grams), and histological tumor type.

Laboratory analyses were performed in the central hospital laboratory using standardized methods and quality control procedures, and results were interpreted according to the local reference ranges. For completeness, the laboratory reference ranges used in our center were as follows: RBC, 4.5–5.9 × 10^6^/µL; WBC, 4–9.5 × 10^3^/µL; neutrophils, 1.8–6.7 × 10^3^/µL; lymphocytes, 0.8–3.8 × 10^3^/µL; eosinophils, 0–0.4 × 10^3^/µL; basophils, 0–0.1 × 10^3^/µL; monocytes, 0.1–0.9 × 10^3^/µL; PLT, 150–400 × 10^3^/µL; ESR, 0–15 mm/h; fibrinogen, 200–393 mg/dL; FT3, 3.54–6.47 pmol/L; FT4, 11.50–22.70 pmol/L; TSH, 0.55–4.78 mIU/L; total protein, 6.4–8.2 g/dL; glycemia, 74–106 mg/dL; and creatinine, 0.7–1.2 mg/dL. Serum TSH, FT3, and FT4 concentrations were measured using automated chemiluminescent immunoassays, in accordance with the manufacturer’s protocols, within the central hospital laboratory. All analyses were performed under routine internal and external quality control procedures, and results were interpreted using the reference intervals applied by the institutional laboratory at the time of testing. Complete blood counts were performed using an automated hematology analyzer in the hospital laboratory, according to standardized operating procedures. Differential leukocyte counts were obtained through automated analysis, with internal quality control performed daily in accordance with laboratory accreditation standards.

Thyroid-specific tumor and autoimmunity markers (serum calcitonin, thyroglobulin (Tg), anti-thyroglobulin antibodies (TgAb), and thyroid peroxidase antibodies (TPOAb)) were not available in a standardized manner for the entire study period and for all patients in this retrospective cohort. These tests were ordered selectively based on clinical suspicion and evolving institutional protocols; therefore, they were not included in the comparative analyses to avoid substantial missing data and selection bias.

**Figure 1:**
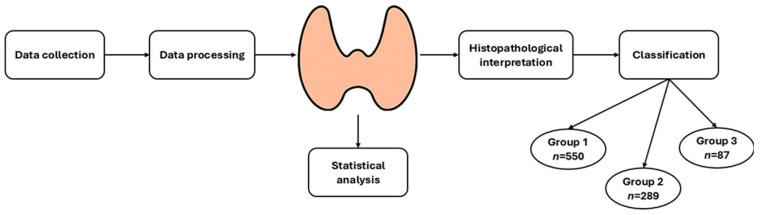
Workflow of the analytical process used in the study, outlining the sequential steps from data collection and preprocessing to histopathological interpretation, statistical evaluation, and final classification of patients into the three predefined histopathological groups.

To provide a clear overview of the methodological framework used in this study, we developed a schematic workflow that summarizes the main analytical steps—from data collection and preprocessing to histopathological evaluation and statistical testing. Figure 1 illustrates the complete pathway applied for integrating clinical, biochemical, hormonal, and histopathological parameters into the final patient classification.

### 2.3. Specimen Preparation and Histopathological Evaluation

All thyroidectomy specimens were processed in the Pathology Department of the “Pius Brînzeu” Emergency County Hospital. The specimens were fixed in 10% neutral-buffered formalin for 24–72 h, and the external surface was inked. The measurements and total weight of each specimen were recorded. After separation of the lobes and isthmus, the dimensions and weight of each component were documented.

Following serial sectioning, we recorded for each nodule the size, capsule thickness, distance to the surgical resection margin, location within the gland, consistency, color, and degree of delineation. The standard practice in our laboratory is to sample at least three sections per lobe plus one section from the isthmus, with a slice thickness of 3–4 mm. For encapsulated nodules, additional blocks including the interface between the capsule and adjacent thyroid parenchyma were submitted. Additional tissue sections were also sampled when a lesion appeared suspicious for malignancy. The relationship between the tumor and the thyroid capsule, as well as potential extension into extrathyroidal soft tissues, was systematically evaluated. All identifiable lymph nodes within the surgical specimen were sampled.

Resected specimens were processed by standard paraffin embedding and stained with hematoxylin and eosin for routine microscopic examination.

Immunohistochemistry: For immunohistochemical (IHC) analysis, additional 3–4 µm sections from selected paraffin blocks were mounted on Super Frost Ultra Plus slides (Thermo Fisher Scientific, Waltham, MA, USA). The primary antibodies used were: TTF1 [clone SPT24, Leica, ready to use (RTU)], CD56 (clone CD564, Leica Microsystems, Wetzlar, Germany, ready-to-use [RTU]), Chromogranin A (clone 5H7, Leica, RTU), D2-40 (clone D2-40, Dako, Glostrup, Denmark, RTU), CD31 (clone JC70A, Leica, RTU), and Synaptophysin (clone 27G12, Leica, RTU). Antigen retrieval was performed by heat-induced epitope retrieval (HIER) in target retrieval solution with pH 6 (for CD56, TTF1, Chromogranin A) or pH 9 (for D2-40, CD31, Synaptophysin), according to the manufacturers’ recommendations. IHC was applied in selected cases to support the differential diagnosis between follicular-patterned lesions, neuroendocrine differentiation, and lymphatic or vascular involvement, based on the routine histological and clinical context. Specifically, TTF1 was used to confirm thyroid epithelial origin, CD56, chromogranin A, and synaptophysin to assess neuroendocrine differentiation (particularly in suspected medullary thyroid carcinoma), while D2-40 and CD31 were employed to evaluate lymphatic and vascular involvement, respectively.

### 2.4. Enrollment Criteria and Group Definitions

To ensure homogeneity of the study population and the accuracy of the histopathological assessment, we included only patients diagnosed with thyroid nodules who underwent total thyroidectomy, had complete clinical, biochemical, hormonal, surgical, and histopathological records available, were aged 18 years or older, and provided written informed consent for the use of their medical data for research purposes. Patients were excluded if they had incomplete medical records, underwent only partial thyroid surgery such as lobectomy, lacked documented informed consent, had a history of prior thyroid surgical interventions, or presented specimens that could not be reliably evaluated histopathologically.

After applying these criteria, 926 patients were finally included in the study. These patients were divided into three groups according to the histopathological findings:

- Group 1: patients with a single benign lesion or combinations of ≥2 benign lesions;
- Group 2: patients with combinations of benign and malignant lesions;
- Group 3: patients with combinations of ≥2 malignant lesions, regardless of the presence of additional benign lesions.

Group allocation was based exclusively on the final histopathological report for the entire thyroid gland, and each patient was assigned to a single group according to the most severe lesion pattern identified.

### 2.5. Statistical Analysis

Statistical analysis was performed in line with the exploratory nature of this retrospective cohort. Continuous variables are presented as medians and interquartile ranges (IQR) due to their non-Gaussian distribution. Differences between two groups were assessed using the Mann–Whitney U test, while comparisons across three groups were performed using the Kruskal–Wallis test. The Mantel–Haenszel chi-square test was applied to compare frequency distributions between groups for categorical variables. Statistical significance was set at *p* < 0.05, and all analyses were conducted using Stata 19.0 (StataCorp, College Station, TX, USA). Given the exploratory design, no formal adjustment for multiple comparisons was applied; consequently, *p*-values should be interpreted as descriptive and hypothesis-generating.

## 3. Results

### 3.1. Study Population

A total of 926 patients who underwent total thyroidectomy with complete histopathological evaluation were included in the final analysis. Group 1 (benign–benign associations) comprised 550 patients (59.4%), Group 2 (benign–malignant associations) included 289 patients (31.2%), and Group 3 (malignant–malignant associations) included 87 patients (9.4%). The median age across the cohort was 56 years (IQR 47–64), with no statistically significant difference between groups (*p* = 0.301). Women represented most cases across all three groups; however, the proportion of female patients differed significantly (*p* < 0.001), with the lowest percentage of women in Group 3 (80.5%) compared to Group 1 (93.3%) and Group 2 (90.7%). Thus, male sex was more common in the multiple-malignant group. No significant group differences were observed regarding urban vs. rural residence (*p* = 0.805).

### 3.2. Clinical and Biochemical Parameters

Most hematological and biochemical parameters did not differ significantly across the three study groups, indicating a generally comparable systemic and metabolic profile. Neutrophil counts showed a statistically significant difference in the overall comparison across the three groups; however, the absolute differences were small (Δ median approximately 0.2–0.4 × 10^3^/µL), and the Group 2 vs. Group 3 comparison was not statistically significant (*p* = 0.071), limiting any potential clinical relevance. All other hematological parameters—including lymphocytes, monocytes, eosinophils, basophils, platelet count, and total leukocyte count—showed no significant intergroup differences (all *p* > 0.05). Similarly, biochemical markers such as creatinine, glycemia, fibrinogen, and serum proteins were evenly distributed, with no statistically significant variation (all *p* > 0.05). Thyroid-related hormonal markers (TSH, FT3, FT4) also demonstrated comparable median levels across the three groups (TSH *p* = 0.122; FT3 *p* = 0.560; FT4 *p* = 0.456), indicating that thyroid function did not differ substantially according to the histopathological profile.

Specimen dimensions (length, width, thickness) and total gland mass were likewise similar among groups, suggesting that morphological gross features alone do not reliably discriminate between benign–benign, benign–malignant, or malignant–malignant lesion associations.

A detailed comparison of demographic characteristics, hematological and biochemical parameters, hormonal profile, and gross morphological features across the three study groups is presented in Table 1. As shown, most baseline clinical and laboratory variables were similarly distributed among the groups, except for neutrophil count, which demonstrated a modest but statistically significant increase in the multiple-malignant group. The full set of parameters and corresponding statistical analyses are summarized below.

### 3.3. Comorbidities

Across the three study groups, the prevalence of major comorbidities—including diabetes mellitus, arterial hypertension, and other benign or malignant tumors—did not differ significantly. Diabetes was present in 10.7% of the overall cohort, with similar proportions in Group 1 (9.3%), Group 2 (13.5%), and Group 3 (10.5%), yielding no significant difference (*p* = 0.165). Arterial hypertension followed a comparable pattern, being identified in approximately half of the patients, with slightly higher rates in Groups 2 and 3 (56.6% and 59.3%, respectively) compared with Group 1 (50.7%), but without reaching statistical significance (*p* = 0.138). Body weight categories, including overweight and obesity classes I–III, also showed no significant variation among the three groups (all *p* > 0.05). Similarly, the presence of other malignant tumors was equally distributed, with no significant differences between groups (*p* = 0.582). Overall, comorbid conditions appeared to be evenly distributed, indicating that the differences observed in pathological outcomes were not attributable to disparities in the baseline health status of the study population.

A detailed comparison of comorbidities and body weight categories across the three groups is presented in Table 2. As shown, the prevalence of diabetes mellitus, arterial hypertension, other benign or malignant tumors, as well as BMI-defined weight categories, was similar among groups, with no statistically significant differences. These findings suggest that baseline metabolic and cardiovascular conditions were evenly distributed and are unlikely to account for the differences observed in the histopathological profiles.

### 3.4. Histopathological Findings

Benign thyroid pathology was frequent across the cohort; however, substantial differences emerged in how these lesions were distributed among the three groups. Multinodular goiter (NG) demonstrated the most pronounced variation, being identified in only 21.6% of Group 1 but reaching 98.9% in Group 3 (*p* < 0.001), and showing similarly significant differences across all three groups (*p* < 0.001). Follicular adenoma also exhibited statistically significant, although less marked, differences between groups (*p* = 0.038 for left lobe; *p* = 0.027 for right lobe).

By study design, Group 1 included exclusively benign–benign histopathological associations, whereas malignant lesions were observed only in Groups 2 and 3, in accordance with the predefined grouping criteria. Accordingly, malignant entities—including papillary thyroid microcarcinoma, papillary thyroid carcinoma, and follicular thyroid carcinoma, together with their respective subtypes—are presented descriptively, without inferential statistical testing involving Group 1. Differences in subtype distribution, multifocality, and lobe involvement were primarily observed within and between Groups 2 and 3, reflecting increasing histopathological complexity across the predefined categories. A detailed summary of the main benign and malignant histopathological categories identified in the three groups is presented in Table 3. As shown, multinodular goiter and follicular adenoma demonstrate significant variation across groups, while all malignant lesions—including papillary thyroid microcarcinoma, papillary thyroid carcinoma, and follicular thyroid carcinoma—follow a markedly different distribution, with increasing prevalence from Group 1 to Group 3. Isthmus involvement also reflects this gradient, further highlighting the escalation of malignant burden across the predefined categories.

### 3.5. Isthmus Lesions

Thyroid lesions originating in the isthmus were less frequent overall and were confined to patients with malignant-associated disease. Papillary thyroid carcinoma arising in the isthmus was absent from Group 1 and identified in both Groups 2 and 3, with higher frequencies observed in Group 3 compared with Group 2; however, this difference did not reach statistical significance (G2 vs. G3 *p* = 0.071).

Papillary thyroid microcarcinomas located in the isthmus followed a similar distribution, being observed exclusively in Groups 2 and 3, without statistically significant differences between these two groups (G2 vs. G3 *p* = 0.503). Rare cases of follicular and medullary thyroid carcinoma arising in the isthmus were also identified in both malignant-associated groups, again without statistically significant differences between Group 2 and Group 3.

The distribution of thyroid lesions originating in the isthmus is summarized in Table 4. Isthmus involvement is presented descriptively for Group 1, which contained no malignant lesions by study design, and inferential statistical analysis is restricted to the clinically relevant comparison between Group 2 and Group 3.

### 3.6. Subgroup Analyses

In addition to the primary comparison across the three histopathological groups, several exploratory subgroup analyses were conducted to further evaluate whether specific benign or malignant entities exhibited distinct clinical, biochemical, or inflammatory patterns that were not captured by the main group-based classification. These secondary analyses included: (1) benign histological subtypes within Group 1; (2) benign–malignant coexistence (Group 2) versus multiple malignant lesions (Group 3); (3) exclusively benign lesions (Group 1) versus extensively malignant disease (Group 3).

Benign Subgroup Analysis (Group 1 Only): Patients with purely benign thyroid disease showed no significant clinical or biochemical differences between multinodular goiter and follicular adenoma. Median WBC, neutrophil, lymphocyte, and platelet counts were comparable between benign subtypes (all *p* > 0.05). Thyroid function tests also demonstrated similar distributions (TSH median 1.9 mIU/L, FT3 median 5.0 pmol/L across benign patterns). All other clinical, hormonal, and biochemical parameters demonstrated *p* > 0.05, indicating uniform systemic profiles across benign lesions.

Benign + Malignant vs. Multiple Malignant (Group 2 vs. Group 3): When comparing Group 2 and Group 3, no significant differences were observed for age (57.0 vs. 54.0 years, *p* = 0.157), neutrophils (4.8 vs. 5.0 × 10^3^/µL, *p* = 0.071), lymphocytes (2.1 vs. 2.0 × 10^3^/µL, *p* = 0.397), platelet count (268 vs. 270 × 10^3^/µL, *p* = 0.972), glycemia (104 vs. 104 mg/dL, *p* = 0.811), creatinine (0.8 vs. 0.8 mg/dL, *p* = 0.548), or TSH (1.9 vs. 1.9 mIU/L, *p* = 0.351).

Similarly, comorbidities—including diabetes (13.5% vs. 10.5%, *p* = 0.454) and hypertension (56.6% vs. 59.3%, *p* = 0.656)—as well as BMI categories showed no statistically significant variation (all *p* > 0.05). Sex was the only demographic parameter with a significant difference, with a higher proportion of males in Group 3 (19.5% vs. 9.3% in Group 2, *p* = 0.009).

A detailed comparison of the malignant histopathological subtypes identified in Group 2 and Group 3 is presented in Table 5. As shown, several aggressive variants—including classical PTC, follicular-variant PTC, tall-cell PTC, Hürthle-cell carcinoma, minimally invasive FTC, and medullary carcinoma—were significantly more prevalent in patients with multiple malignant lesions, highlighting the distinct histopathological complexity characteristic of Group 3 (all *p* > 0.05).

Benign vs. Multiple Malignant (Group 1 vs. Group 3): A statistically significant reduction in lymphocyte count was observed in Group 3 compared with Group 1 (2.0 [1.7–2.4] vs. 2.1 [1.6–2.5] × 10^3^/µL; *p* = 0.009), suggesting a possible immune alteration associated with extensive malignant disease. Neutrophil count showed a nonsignificant upward trend (5.0 vs. 4.6 × 10^3^/µL, *p* = 0.116). Age (54 vs. 56 years, *p* = 0.301), glycemia (104 vs. 104 mg/dL, *p* = 0.829), creatinine (0.8 vs. 0.8 mg/dL, *p* = 0.193), TSH (1.9 vs. 1.9 mIU/L, *p* = 0.122), FT3 (5.0 vs. 5.0 pmol/L, *p* = 0.560), and thyroid gross morphology (all *p* > 0.05) remained comparable.

The comparative distribution of benign and malignant histopathological categories between Group 1 and Group 3 is summarized in Table 6. All malignant entities—including PTMC, PTC, FTC, and aggressive variants—were exclusively identified in Group 3 (all *p* < 0.001), while multinodular goiter was significantly more frequent in this group as well, supporting the role of multinodular architecture as a substrate for multifocal malignant transformation.

Overall, across subgroup analyses, systemic laboratory markers showed only minimal variation between patients with different histopathological patterns. The observed differences in histological composition reflect the predefined group classification and provide descriptive characterization rather than independent predictive findings.

## 4. Discussion

The present study highlights that systemic biochemical and hormonal parameters show minimal discriminatory power between benign and malignant thyroid nodules, while histopathological evaluation provides detailed post hoc characterization of lesion coexistence, multifocality, and subtype distribution within the predefined groups. This finding is consistent with previous reports showing that thyroid function tests such as TSH, FT3, and FT4 often fail to differentiate malignant from benign nodules, despite earlier hypotheses suggesting a possible association between elevated TSH and increased malignancy risk [31]. Our results reinforce the notion that biochemical markers alone are insufficient for risk stratification and should be interpreted in conjunction with imaging and histopathology.

Consistent with previous studies, most demographic and clinical characteristics—including age, BMI, comorbidities, and metabolic parameters—did not differ significantly between patient groups [31]. This aligns with prior evidence suggesting that classical metabolic indicators, such as glycemia or renal function markers, have limited utility in predicting thyroid malignancy. Similarly, serum proteins, fibrinogen, and routine hematological indices were evenly distributed among groups, reinforcing the notion that systemic inflammatory or metabolic dysregulation is not a defining feature of malignant thyroid disease [32]. The only exception observed in our cohort was the modest increase in neutrophil count and the relative reduction in lymphocyte count among patients with multiple malignant lesions [33]. Although statistically significant, these differences were small and likely insufficient to serve as standalone diagnostic markers. Nonetheless, this pattern may reflect subtle immune alterations accompanying multifocal or more biologically aggressive disease, as previously suggested in studies linking neutrophil–lymphocyte dynamics with oncogenic processes.

In contrast, histopathological evaluation documented distinct patterns of lesion coexistence and subtype distribution that reflect the predefined group classification. As expected, malignant lesions—including PTMC, PTC, and FTC—were strictly confined to Groups 2 and 3, with Group 3 demonstrating a substantially higher prevalence of multifocal involvement and aggressive variants such as tall-cell, oncocytic, and hobnail subtypes. These observations are consistent with the literature, which recognizes multifocality, variant histology, and isthmus involvement as key predictors of more advanced or clinically challenging disease [34]. The markedly increased frequency of multinodular goiter in patients with multiple malignant lesions (98.9%) further supports the hypothesis that nodular architectural complexity may facilitate or reflect underlying malignant transformation.

Histopathological analysis demonstrated the distribution of malignant entities across the predefined groups, with multifocal and variant patterns being confined to the malignant-dominant category by study design. This aligns with large retrospective studies demonstrating that papillary carcinoma is the most prevalent thyroid malignancy, accounting for nearly 85–90% of cases [35]. Moreover, aggressive histological subtypes such as tall-cell and hobnail variants have been repeatedly associated with poorer prognosis and higher recurrence rates [36]. Our findings confirm that these variants cluster in patients with multifocal malignant disease, underscoring the importance of detailed histopathological evaluation in surgical specimens.

Interestingly, multinodular goiter (MNG) was strongly associated with malignant lesions, being present in nearly all patients with multiple malignant nodules. This observation supports prior evidence that multinodularity does not preclude malignancy and may, in fact, coexist with papillary microcarcinomas [37]. Clinicians should therefore avoid assuming benignity in multinodular thyroid disease and maintain vigilance for occult carcinoma, particularly in surgical candidates.

The lack of significant differences in comorbidities such as diabetes mellitus, hypertension, and obesity across groups suggests that baseline metabolic and cardiovascular conditions do not substantially influence thyroid malignancy risk. This is consistent with prior epidemiological studies that failed to establish strong causal links between metabolic syndrome and thyroid cancer incidence [32,38,39]. Instead, genetic predispositions (e.g., *BRAF*, *RAS* mutations) and environmental exposures remain the dominant risk factors [31,40].

From a clinical perspective, our results emphasize that histopathological examination remains essential for definitive diagnosis, while routinely available systemic biochemical and hormonal markers showed limited ability to parallel postoperative histopathological patterns. This supports current American Thyroid Association (ATA) guidelines, which prioritize ultrasound risk stratification and fine-needle aspiration (FNA) cytology, supplemented by molecular testing when necessary [31,41]. Future research should prioritize multimodal risk stratification approaches; in our cohort, the small between-group differences in neutrophil counts were not clinically informative and should be regarded as exploratory. The absence of significant differences in thyroid function markers (TSH, FT3, FT4) between groups is noteworthy. Although some prior studies have proposed a potential association between elevated TSH levels and increased malignancy risk [42], our dataset demonstrated comparable hormonal profiles across all categories of disease. This discrepancy may reflect population-specific differences, the retrospective design of the study, or the multifactorial interplay between thyroid autoimmunity, iodine status, and genetic predisposition [43]. Taken together, the results reinforce the conclusion that hormonal markers, while essential for functional assessment, do not reliably distinguish benign from malignant nodular disease, consistent with findings from large retrospective cohorts [31].

Another key finding of this study is the distribution of malignancies arising in the thyroid isthmus. Although relatively uncommon, isthmus-originating cancers were significantly more frequent in Groups 2 and 3, echoing previous reports that associate isthmus location with more aggressive clinicopathological features [44]. Whether this reflects anatomical considerations, lymphatic drainage patterns, or intrinsic biological differences remains a topic for future investigation, as suggested by studies highlighting the unique behavior of isthmus tumors compared with lobar counterparts [45].

Subgroup analyses provided further refinements to our understanding of disease behavior. Patients with benign-only lesions exhibited no clinically meaningful biochemical or inflammatory differences between multinodular goiter and follicular adenoma, supporting the homogeneity of benign pathology at the systemic level [32]. Comparisons between Group 2 and Group 3 revealed major histopathological distinctions despite largely similar clinical profiles, highlighting multifocal malignant transformation as the defining feature of extensive disease rather than patient-related systemic factors [31]. Likewise, the presence of aggressive variants exclusively in the multiple-malignant group reinforces their strong association with more advanced pathological progression, consistent with prior evidence that tall-cell, oncocytic, and hobnail variants are linked to poorer outcomes [34].

Taken together, these findings indicate that routinely available clinical, biochemical, and hormonal parameters do not reliably reflect the histopathological patterns identified postoperatively in patients with thyroid nodules. This underscores the importance of comprehensive pathological evaluation, particularly in cases with multinodular architecture or suspected multifocal disease. While routine laboratory markers remain indispensable for perioperative and metabolic assessment, their diagnostic performance in malignancy risk stratification appears limited.

The strengths of this study include the large sample size, the comprehensive evaluation of all patients undergoing total thyroidectomy, and the uniform pathological assessment performed within a single tertiary center. However, several limitations deserve consideration. The retrospective design introduces inherent selection and information biases, and the lack of molecular testing limits deeper insights into tumor biology and mutation-specific behavior. Furthermore, although the study population was homogeneous with respect to regional and clinical characteristics, external generalizability should be approached with caution.

This study has several limitations that should be acknowledged. An important limitation is the unavailability of standard thyroid tumor and autoimmunity biomarkers—particularly serum calcitonin, Tg, TgAb, and TPOAb—in a complete and uniform manner across the entire cohort. Because these assays were performed selectively in routine practice (e.g., in patients with clinical or imaging suspicion), including them would have introduced substantial missingness and potential selection bias. This limitation is especially relevant given that medullary thyroid carcinoma (MTC) cases were included, for which calcitonin is a key preoperative biomarker; therefore, our findings should not be interpreted as assessing or excluding the diagnostic value of these thyroid-specific markers. Therefore, the present study should not be interpreted as a comprehensive biochemical characterization of thyroid nodules, but rather as an evaluation of routinely available systemic laboratory parameters used in everyday clinical practice. First, its retrospective design inherently introduces selection and information biases, as only patients who underwent total thyroidectomy and had complete histopathological evaluation were included, potentially overrepresenting more complex or symptomatic cases. Second, the analysis relied exclusively on clinical, biochemical, hormonal, and morphological parameters available in routine practice; advanced molecular profiling, which could have provided deeper insights into tumor biology and malignant potential, was not systematically performed. Third, although laboratory tests were standardized within a single tertiary center, inter-individual variability in preoperative factors—such as iodine intake, autoimmune status, or medication use—could not be fully controlled. Additionally, the absence of long-term postoperative follow-up limits the interpretation of clinical impact, recurrence risk, or prognostic implications of the observed histopathological patterns. Finally, because the study population originated from a single regional institution, external generalizability to other demographic or geographic populations may be limited, warranting cautious extrapolation of the findings.

Future research should aim to integrate molecular diagnostics, advanced imaging features, and immune-inflammatory profiling to refine the predictive models for thyroid malignancy. Prospective validation of combined clinical–molecular–histological algorithms may offer improved accuracy in identifying patients at risk for multifocal or aggressive disease patterns, ultimately supporting more personalized surgical and postoperative management strategies.

## 5. Conclusions

This seven-year retrospective analysis demonstrates that routinely available clinical, biochemical, and hormonal parameters show limited ability to discriminate between patients with different histopathological patterns of thyroid nodular disease. While histopathological evaluation remains the definitive diagnostic standard, the present findings indicate that systemic laboratory markers commonly used in clinical practice do not reliably reflect underlying histopathological complexity. These results underscore the need for multimodal diagnostic approaches integrating imaging, cytology, and molecular profiling to improve preoperative risk stratification.

## Figures and Tables

**Table 1:** Comparison of demographic, clinical, biochemical, hormonal, and gross morphological parameters across the three histopathological groups. Continuous variables are reported as median (interquartile range) and were analyzed using the Kruskal–Wallis non-parametric test due to their non-normal distribution. Categorical variables are presented as number (percentage) and were analyzed using the Mantel–Haenszel chi-square test. A two-sided *p* value < 0.05 was considered statistically significant. Continuous data are presented as median (interquartile range). Overall *p*-values were calculated using the Kruskal–Wallis test. Pairwise comparisons (Group 1 vs. Group 2, Group 1 vs. Group 3, Group 2 vs. Group 3) were performed using the Mann–Whitney U test.

Parameter	Group 1 (*n* = 550)	Group 2 (*n* = 289)	Group 3 (*n* = 87)	Overall *p*-Value	G1 vs. G2 *p*	G1 vs. G3 *p*	G2 vs. G3 *p*
Age (years)	56.0 (48.0–64.0)	57.0 (47.0–65.0)	54.0 (45.0–63.0)	0.301	0.287	0.086	0.157
Sex, female, n (%)	513 (93.3%)	262 (90.7%)	70 (80.5%)	<0.001	0.174	0.0003	0.013
Residence, urban, n (%)	373 (67.8%)	193 (66.8%)	56 (64.4%)	0.805	0.757	0.540	0.699
Normal weight, n (%)	138 (25.1%)	68 (23.5%)	21 (24.1%)	0.879	0.673	0.895	0.887
Hematological parameters							
Platelet count, ×10^3^/µL	269.2 (228.0–306.0)	268.0 (229.0–296.0)	270.0 (218.0–312.0)	0.989	0.891	0.367	0.972
Platelet distribution width (PDW)	28.0 (12.5–44.0)	27.4 (12.5–48.0)	17.6 (11.6–43.7)	0.540	0.782	0.932	0.279
RBC, ×10^6^/µL	4.6 (4.4–4.9)	4.7 (4.5–4.9)	4.6 (4.5–4.9)	0.364	0.161	0.318	0.710
WBC, ×10^3^/µL	7.6 (6.2–8.7)	7.6 (6.5–8.7)	7.7 (6.4–8.9)	0.200	0.241	0.607	0.412
Neutrophils, ×10^3^/µL	4.6 (3.6–5.7)	4.8 (3.9–5.7)	5.0 (3.9–6.2)	0.021	0.174	0.116	0.071
Lymphocytes, ×10^3^/µL	2.1 (1.6–2.5)	2.1 (1.7–2.6)	2.0 (1.7–2.4)	0.662	0.536	0.009	0.397
Eosinophils, ×10^3^/µL	0.1 (0.1–0.2)	0.1 (0.1–0.2)	0.1 (0.1–0.2)	0.792	0.500	0.603	0.712
Basophils, ×10^3^/µL	0.0 (0.0–0.1)	0.0 (0.0–0.1)	0.0 (0.0–0.1)	0.341	0.146	0.986	0.703
Monocytes, ×10^3^/µL	0.5 (0.3–0.6)	0.5 (0.3–0.6)	0.5 (0.3–0.7)	0.816	0.557	0.614	0.990
Biochemical parameters							
Glycaemia, mg/dL	104.0 (95.0–110.0)	104.0 (94.0–114.0)	104.0 (97.0–112.0)	0.829	0.665	0.710	0.811
Creatinine, mg/dL	0.8 (0.7–0.8)	0.8 (0.7–0.9)	0.8 (0.7–0.9)	0.193	0.071	0.607	0.548
Fibrinogen, mg/dL	351.0 (298.0–389.0)	349.0 (303.0–403.0)	354.0 (289.0–399.0)	0.535	0.425	0.600	0.688
Serum proteins, g/dL	6.9 (6.5–7.4)	6.9 (6.5–7.4)	6.8 (6.5–7.4)	0.728	0.566	0.347	0.793
Hormonal profile							
TSH, mIU/L	1.9 (0.9–1.9)	1.9 (0.9–2.0)	1.9 (1.3–2.1)	0.122	0.225	0503	0.351
FT3, pmol/L	5.0 (4.7–5.3)	5.0 (4.7–5.4)	5.0 (4.6–5.3)	0.560	0.974	0.056	0.306
FT4 (pmol/L)	14.9 (12.3–17.0)	14.8 (12.5–16.8)	14.7 (12.1–16.3)	0.456	0.894	0.042	0.204
Gross morphology							
Thyroid length, cm	7.0 (5.5–8.5)	7.0 (5.5–9.0)	6.5 (5.5–8.0)	0.401	0.731	0.303	0.190
Thyroid width, cm	5.0 (4.0–6.5)	5.0 (4.0–6.5)	4.7 (4.0–6.0)	0.183	0.608	0.223	0.161
Thyroid thickness, cm	3.0 (2.0–4.0)	2.7 (2.0–4.0)	2.8 (2.0–3.7)	0.667	0.466	0.064	0.852
Thyroid mass, g	35.0 (20.0–41.0)	29.0 (19.0–40.0)	27.0 (19.0–39.0)	0.109	0.114	0.499	0.555

**Table 2:** Prevalence of comorbidities and body weight categories across the three histopathological groups. Categorical data are presented as number (percentage). Overall *p*-values were calculated using the chi-square test. Pairwise comparisons between groups (Group 1 vs. Group 2, Group 1 vs. Group 3, Group 2 vs. Group 3) were performed using Fisher’s exact test, as appropriate.

Parameter	Group 1 (*n* = 550)	Group 2 (*n* = 289)	Group 3 (*n* = 87)	Overall *p*-Value	G1 vs. G2 *p*	G1 vs. G3 *p*	G2 vs. G3 *p*
Diabetes mellitus	51 (9.3%)	39 (13.5%)	9 (10.5%)	0.165	0.078	0.696	0.582
Arterial hypertension	279 (50.7%)	163 (56.6%)	51 (59.3%)	0.138	0.127	0.204	0.805
Other benign tumors	50 (9.1%)	35 (12.1%)	7 (8.1%)	0.321	0.186	1.000	0.337
Other malignant tumors	30 (5.5%)	12 (4.2%)	3 (3.5%)	0.582	0.506	0.504	1.000
Normal weight	138 (25.1%)	68 (23.5%)	21 (24.1%)	0.879	0.673	0.895	0.887
Overweight	225 (41.0%)	119 (41.2%)	39 (44.8%)	0.749	0.941	0.558	0.620
Obesity class I	130 (23.6%)	70 (24.2%)	18 (20.7%)	0.812	0.865	0.588	0.565
Obesity class II	42 (7.6%)	25 (8.7%)	5 (5.7%)	0.553	0.595	0.662	0.500
Obesity class III	15 (2.7%)	7 (2.4%)	4 (4.6%)	0.420	1.000	0.313	0.287

**Table 3:** Distribution of main benign and malignant histopathological findings across the three study groups. Benign entities were compared using inferential statistics across groups. For malignant lesions, Group 1 contained no cases by study design; therefore, these data are presented descriptively, and inferential statistical testing is restricted to clinically relevant comparisons between Group 2 and Group 3 (chi-square or Fisher’s exact test, as appropriate).

Histopathological Category	Group 1 (*n* = 550)	Group 2 (*n* = 289)	Group 3 (*n* = 87)	G2 vs. G3 *p*
Multinodular goiter (NG)	119 (21.6%)	202 (69.9%)	86 (98.9%)	<0.001
Follicular adenoma (FA)	68 (12.3%)	52 (18.0%)	9 (10.3%)	0.027
Papillary thyroid microcarcinoma (PTMC, all)	0 (0%)	90 (31.1%)	27 (31.0%)	0.983
Papillary thyroid carcinoma (PTC, all)	0 (0%)	108 (37.3%)	54 (62.1%)	0.001
Follicular thyroid carcinoma (FTC, all)	0 (0%)	14 (4.8%)	10 (11.5%)	0.049
Medullary thyroid carcinoma (MTC)	0 (0%)	3 (1.0%)	2 (2.3%)	0.612
PTMC–isthmus	0 (0%)	16 (5.5%)	7 (8.0%)	0.503
PTC–isthmus	0 (0%)	14 (4.8%)	10 (11.5%)	0.071

**Table 4:** Prevalence of papillary, follicular, and medullary thyroid carcinoma arising in the isthmus across the three study groups. Data are presented as number (percentage). Pairwise comparisons were performed exclusively between Group 2 and Group 3 using Fisher’s exact test, as appropriate. Group 1 contained no malignant lesions by study design and is presented descriptively. Abbreviations: PTMC, papillary thyroid microcarcinoma; PTC, papillary thyroid carcinoma; FTC, follicular thyroid carcinoma; MTC, medullary thyroid carcinoma.

Parameter	Group 2 (*n* = 289)	Group 3 (*n* = 87)	G2 vs. G3 *p*
PTMC–isthmus	16 (5.5%)	7 (8.0%)	0.503
PTC–isthmus	14 (4.8%)	10 (11.5%)	0.071
FTC–isthmus	2 (0.7%)	1 (1.1%)	1.000
MTC–isthmus	1 (0.3%)	1 (1.1%)	0.437

**Table 5:** Comparative prevalence of malignant histopathological subtypes between Group 2 (benign + malignant lesions) and Group 3 (multiple malignant lesions). Values are presented as number (percentage), and statistical significance was assessed using the Mantel–Haenszel chi-square test, with *p* < 0.05 considered statistically significant. Abbreviations used in the table include: PTC, papillary thyroid carcinoma; FV-PTC, follicular-variant papillary thyroid carcinoma; FTC, follicular thyroid carcinoma; MI-FTC, minimally invasive follicular thyroid carcinoma.

Histopathological Variant	Group 2	Group 3	*p*-Value
Classical PTC (all lobes)	108 (37.3%)	54 (62.1%)	<0.001
FV-PTC (Right lobe)	17 (5.9%)	9 (10.3%)	0.046
MI-FTC (Right lobe)	2 (0.7%)	3 (3.4%)	0.011
Hurthle-cell carcinoma (Right lobe)	3 (1.0%)	4 (4.6%)	0.011
Tall-cell PTC (Left lobe)	3 (1.0%)	4 (4.6%)	0.049
Tall-cell PTC (Right lobe)	3 (1.0%)	5 (5.7%)	0.002
Medullary thyroid carcinoma	3 (1.0%)	2 (2.3%)	0.049

**Table 6:** Comparative distribution of benign and malignant histopathological categories between Group 1 (benign) and Group 3 (multiple malignant lesions). Values are expressed as number (percentage). Statistical significance was evaluated using the Mantel–Haenszel chi-square test (*p* < 0.05).

Histopathological Category	Group 1	Group 3	*p*-Value
Multinodular goiter (NG)	119 (21.6%)	86 (98.9%)	<0.001
Papillary thyroid microcarcinoma (PTMC)	0 (0%)	27 (31.0%)	<0.001
Papillary thyroid carcinoma (PTC, all subtypes)	0 (0%)	54 (62.1%)	<0.001
Follicular thyroid carcinoma (FTC, all subtypes)	0 (0%)	10 (11.5%)	<0.001
Aggressive PTC variants (hobnail, oncocytic, tall-cell)	0 (0%)	Present	<0.001

## Data Availability

The original contributions presented in this study are included in the article. Further inquiries can be directed to the corresponding author.

## Abbreviations

The following abbreviations are used in this manuscript:

AJCC	American Joint Committee on Cancer
ATA	American Thyroid Association
ATC	Anaplastic Thyroid Carcinoma
BMI	Body Mass Index
CBC	Complete Blood Count
CD31	Cluster of Differentiation 31 (vascular marker)
CD56	Cluster of Differentiation 56
D2-40	Podoplanin antibody (lymphatic marker)
ESR	Erythrocyte Sedimentation Rate
FA	Follicular Adenoma
FNA	Fine-Needle Aspiration
FTC	Follicular Thyroid Carcinoma
FV-PTC	Follicular Variant Papillary Thyroid Carcinoma
FT3	Free Triiodothyronine
FT4	Free Thyroxine
H&E	Hematoxylin and Eosin
HIER	Heat-Induced Epitope Retrieval
Huc-TC	Hürthle Cell (Oncocytic) Thyroid Carcinoma
IHC	Immunohistochemistry
IQR	Interquartile Range
LL	Left Lobe
MNG	Multinodular Goiter
MI-FTC	Minimally Invasive Follicular Thyroid Carcinoma
MTC	Medullary Thyroid Carcinoma
NLR	Neutrophil-to-Lymphocyte Ratio
NG	Nodular Goiter / Multinodular Goiter
PDW	Platelet Distribution Width
PLT	Platelets
PTC	Papillary Thyroid Carcinoma
PTMC	Papillary Thyroid Microcarcinoma
RL	Right Lobe
RBC	Red Blood Cell Count
Stata	Statistical Analysis Software (StataCorp)
TLA	Three-Letter Acronym
TC-PTC	Tall-Cell Variant Papillary Thyroid Carcinoma
TP	Total Protein
TSH	Thyroid Stimulating Hormone
TTF1	Thyroid Transcription Factor 1
US	Ultrasound
WBC	White Blood Cell Count
WHO	World Health Organization
